# Demographics of Total Hip and Knee Arthroplasty in Nepal: An Observational Study

**DOI:** 10.31729/jnma.8821

**Published:** 2024-11-30

**Authors:** Ishwor Ghimire Padhya, Suman Subedi, Prakash Gyawali, Sushant Regmi

**Affiliations:** 1 Department of Orthopedics, Pokhara Academy of Health Sciences, Pokhara, Nepal; 2 Gorkha District Hospital, Gorkha, Nepal; 3 Nepal Orthopedic Hospital, Jorpati, Kathmandu, Nepal; 4 Department of General Practice and Emergency Medicine, Patan Academy of Health Sciences, Lagankhel, Lalitpur, Nepal

**Keywords:** *arthroplasty*, *demography*, *hip replacement*, *knee replacement*, *Nepal*

## Abstract

**Introduction:**

Hip and knee arthroplasty surgeries are essential for treating end-stage osteoarthritis, providing significant functional improvements. Despite a global rise in these procedures, Nepal lacks a National Joint Registry, resulting in a gap in tracking the exact number of surgeries, outcomes, and complications. This study aims to address this gap regarding the demographics of hip and knee arthroplasties in Nepal.

**Methods:**

An observational study was conducted after obtaining the ethical approval from the Nepal Health Research Council (Reference number: 2024/136) with data collected from prosthesis supplier. The study included all primary hip and knee arthroplasties performed in 2022 and 2023, excluding revision cases and those requiring mega prostheses. Data analysis was performed using Microsoft Excel for descriptive statistics.

**Results:**

There were a total of 1259 surgeries, 916 (72.77%) total knee replacements were performed, with a female predominance of 741 (81.42%) and a median age of 67 years(IQR: 62-71). There were 343 (27.23%) total hip replacements surgeries of which 200 (58.31%) were done in males. Most surgeries 1148 (91.18%) occurred within the Kathmandu Valley.

**Conclusions:**

Total knee arthroplasty accounted for the majority of cases, showing a predominance of female patients with the majority of surgeries in the capital city.

## INTRODUCTION

Hip and knee arthroplasty surgeries are the gold standard for the treatment of end-stage osteoarthritis.^1^ These procedures have become more common globally, and nationally offering improved functional outcomes for the patients.^2-5^ This number is expected to rise by 176% for hip arthroplasty and 139% for knee arthroplasty by 2040.^6^

The exact number of these surgeries performed in Nepal is unclear, as there is no National Joint Registry to track them which creates a significant gap in knowledge regarding the true extent and nature of arthroplasty procedures. This lack of comprehensive data limits our understanding of clinical outcomes, complications, and implant failures, which are crucial for improving patient care.

Previous studies have had the limitation of limited generalizability and have recommended conducting studies with larger sample sizes.^3,7^ This study aims to fill this gap focusing on the total number of hip and knee arthroplasties, gender distribution, and geographical location of surgeries.

## METHODS

This was a descriptive study conducted based on the retrospective data in Nepal with ethical approval from the Nepal Health Research Council (NHRC) (Reference number: 2024/136). Authorization to use data obtained from the implant supplier was also secured and subsequently approved by the Nepal Health Research Council.

The dataset included information collected from patients undergoing arthroplasty, ensuring data was available for potential future needs, such as revision surgeries. To address potential limitations with secondary data reliability, the data underwent subsequent steps which involved examining the dataset for outliers, inconsistencies, and irregularities in distribution. Additionally, cross-verification with data custodians was done by the principal investigator to resolve any discrepancies. The study included all patients who underwent primary total knee arthroplasty (TKA) or total hip arthroplasty (THA) from January, 2022 to December, 2023 with implants supplied by the supplier. Cases involving revision surgeries, tumor-related procedures requiring mega prostheses, and other types of joint arthroplasties were excluded.

Collected data included patient age, gender, laterality of the site of surgery and geographical site for surgery. The study exclusively considered patient data, excluding procedural details such as surgical techniques and postoperative care protocols. Data were securely stored in password-protected systems accessible only to the research team. Initially compiled in Microsoft Excel, the dataset underwent cleaning. Descriptive statistics (mean, median, standard deviation) and frequency distribution analyses were then performed in Excel.

## RESULTS

There were a total of 1259 arthroplasties in the study duration of which 916 (72.76%) were TKR surgeries and 343 (27.24%) were THR surgeries. Among all arthroplasties, 884 (70.21%) surgeries were conducted in females. The median age for patients undergoing TKR was 67 years (IQR: 62-71) with the youngest patient being a 23-year-old male, and the oldest an 88-year-old female. For total hip replacement (THR), 143 (41.69%) cases were documented in 2022, with 200 (58.31%) males of all THR cases. The median age for patients undergoing THR surgery was 55 (IQR 44-64) years ranging from an 18-year-old male to an 82-year-old male. Of all arthroplasties, only 111 (8.82%) were performed outside the Kathmandu valley (Table 1).

**Table 1:** Demographic profile of toal hip and Knee arthroplasties (N=1259).

Surgery	Year	Gender		Side		Location		Total cases n (%)
		Female n (%)	Male n (%)	Left n (%)	Right n (%)	Bilateral n (%)	Outside Kathmandu Valley n (%)		Inside Kathmandu Valley n (%)
TKA (n=916)	2022	298 (32.53)	68 (7.42)	193 (21.07)	160 (17.47)	13 (1.42)	13 (1.42)	353 (38.54)	366 (39.96)
	2023	443 (48.36)	107 (11.68)	233 (25.44)	273 (29.80)	44 (4.80)	53 (5.79)	497 (54.26)	550 (60.04)
THA (n=343)	2022	61 (17.78)	82 (23.91)	65 (18.95)	78 (22.74)	-	17 (4.96)	126 (36.73)	143 (41.69)
	2023	82 (23.91)	118 (34.40)	84 (24.49)	116 (33.82)	-	28 (8.16)	172 (50.15)	200 (58.31)
Total Cases		884 (70.21)	375 (29.79)	575 (45.67)	627 (49.80)	57 (4.53)	111 (8.82)	1148 (91.18)	1259 (100)

## DISCUSSION

In our study, we analyzed 1,259 cases of joint replacement surgeries in Nepal, of which 916 (72.76%) were total knee replacements (TKR) and 343 (27.24%) were total hip replacements (THR). This is a larger sample size than previous reports from Nepal.^2,3^ A study from 2008 to 2015 recorded only 40 hip replacements at a single center, likely reflecting the early days of hip replacement surgeries in the country.^2^ Another single-center study from 2016 to 2020 found 73 hip and knee replacements combined.^3^ Our larger sample likely results from gathering data from suppliers who provide implants to multiple hospitals across Nepal, rather than limiting the data to one center. Hip replacement surgeries has been cited as the "surgery of the century"^8^ and this along with other joint replacement surgeries are expected to grow significantly due to aging populations and rising cases of osteoarthritis.^9^ TKR and THR are considered essential for managing arthritis, with projections suggesting THR could increase by 659% and TKR by 469% by 2060.^6^ Osteoarthritis itself has increased by 132.2% since 1990, and by 2050, knee and hip arthritis are expected to rise by around 75% and 79%, respectively.^9^ In China, for instance, THR surgeries rose 2.4 times, and TKR surgeries increased 5.9 times from 2011 to 2019.^10^ Although our study does not track changes over time, our findings reflect the growing demand for joint replacement surgeries in Nepal, which is in line with global trends.

Our study found higher number of arthroplasties in the year 2023 compared to 2022. It could either be due to a normal increasing trend as we do not have combined data for 2021 or it could be due to the effect of COVID-19 in the year 2022 and the delay in seeking and delivering care. A similar issue was seen in Europe due to late appointments and financial issues.^11^ We found that the median age ofTHR is about 53.02 years and the mean age of TKR is 66.25 years in our center. This age for TKA aligns with the people undergoing arthroplasty in the developed part of the world. A study in the UK which included 2850 patients underwent total knee replacement at the mean age of 66.8 years comparable to ours; however, the mean age of THR was 67.5 years.^12^ This finding could be attributed to the fact that the most common indication ofTHR in developed parts is primary OA (which occurs in the elderly population) while in the developing world, trauma and infection (which commonly occur in the younger population).^13^

In our study, the number of females undergoing total joint replacement was 884 (70.21%) which was much higher than males. Another study among the Nepalese population also reported similar findings as ours where the males were outnumbered by females 35 (66%).^3^ A similar study in the USA consisting of more than 2,552,815 patients also showed higher TKR and THR rates among females compared to males.^11^ To our surprise, 91.18% of patients who underwent joint replacement surgery were treated in the Kathmandu Valley. This high concentration may reflect the level of trust patients place in the capital, where superspecialized services are more readily available. The Kathmandu Valley, which includes the capital city and other nearby cities, houses most of Nepal's major healthcare centers. Although our study did not record patients' home addresses, we recognize that the decision to undergo surgery is shaped by several broader factors, such as the diffusion of technology, availability of specialists, local training standards, financial incentives, and regulatory conditions, which vary widely across regions and countries.^14^ Additionally, it's worth considering that our data source may also be influenced by its location in the Kathmandu Valley, potentially leading to a natural bias in regional coverage.

This study, based on secondary data supplied by implant suppliers, comes with certain limitations. Secondary data can sometimes limit data accuracy, as it may lack completeness or uniformity in recording practices, which we tried address through careful validation and cross-checking. Since data were solely provided by the implant suppliers, there is a possibility that cases from additional suppliers may have been missed, potentially leading to underrepresentation. Additionally, as these suppliers are predominantly located in the Kathmandu Valley, a regional bias may exist, which may affect the generalizability of our findings to more remote areas in Nepal. Furthermore, our exclusion of revision surgeries and cases requiring mega prostheses may also limit the comprehensiveness of the dataset. Establishing a National Joint Registry could overcome many of these limitations, as it would allow centralized data collection, tracking of all joint replacements, and provide a more accurate and inclusive representation of joint replacement trends nationwide.

We strongly recommend the development of a National Joint Registry in Nepal to enable systematic tracking of patient demographics, implant performance, and surgical outcomes across all regions. Such a registry should aim to track patient data, assess the performance and durability of joint implants, monitor surgical outcomes at hospitals, and evaluate the performance of surgeons performing the procedures.^15^ They also allow case identification in the event of a product recall or adverse surgical outcomes, provide insight into practice variation, and be a valuable resource for further research.^16,17^ If a national registry is not feasible in the short term, more coordinated efforts among suppliers and hospitals to standardize and share data would improve the robustness of future studies. Despite these limitations, a strength of our study is that it captures a substantially larger sample of cases compared to previous studies, providing a foundational demographic overview of hip and knee arthroplasty trends in Nepal.

## CONCLUSIONS

Total knee arthroplasty accounted for the majority of cases, showing a predominance of female patients. Conversely, males constituted the majority in total hip replacement surgeries. A substantial portion of these surgeries took place within Kathmandu Valley.
